# Use of Only Oral Rehydration Salt Solution for Successful Management of a Young Infant with Serum Sodium of 201 mmol/L in an Urban Diarrhoeal Diseases Hospital, Bangladesh

**DOI:** 10.3329/jhpn.v30i3.12301

**Published:** 2012-09

**Authors:** Mohammod J. Chisti, Mark A.C. Pietroni, Mohammad Samsul Alom, Jonathan Harvey Smith

**Affiliations:** ^1^icddr,b, GPO Box 128, Dhaka 1000, Bangladesh; ^2^Portex Department of Paediatric Anaesthesia, Institute of Child Health, UCL, London, UK

**Keywords:** Diarrhoea, Hypernatraemia, Infant, ORS, Bangladesh

## Abstract

A boy aged 4 months 7 days was admitted to the Intensive Care Unit (ICU) of the Dhaka Hospital of icddr,b, Dhaka, Bangladesh, with the problems of acute watery diarrhoea with some dehydration, pneumonia, lethargy, and hypernatraemia (serum sodium of 201 mmol/L). Correction for hypernatraemia was tried by using only oral rehydration salt (ORS) solution. Seizures occurred during correction of the hypernatraemia. These were difficult to control and required three doses of injection lorazepam, a loading dose of injection phenobarbitone, followed by injection phenytoin and finally two doses of injection mannitol (even though there was no clinical or imaging evidence by ultrasonography or computed tomography of cerebral oedema). The correction was continued with ORS, and all the anticonvulsants were successfully weaned without any further seizures, and the patient recovered without any overt neurological sequelae. We present a case report of extreme hypernatraemia, which was successfully managed using only ORS.

## INTRODUCTION

Hypernatraemia is a serious ramification of diarrhoea often associated with death (1). Death may occur either due to hypernatraemia itself or anytime during its correction. Hypernatraemia causes widespread cerebral haemorrhage, thromboses, and subdural effusions, leading to permanent neurologic deficit and death (2,3). On the other hand, improper and aggressive rehydration in patients with prolonged hyperosmolality has been shown to result in a rapid fall in extracellular fluid (ECF) osmolality, leading to cerebral oedema and death (4,5). Ideally, correction should result in a fall of serum sodium of around 10-12 mmol/L/24 hours, preventing cerebral oedema and convulsion (6,7). However, when hypernatraemia develops over a period of few hours, a rapid correction at a rate of 1.0 mmol/L/hour prevents the risk of cerebral oedema because accumulated electrolytes are rapidly extruded from the brain cells (8,9). The choice of fluid for the correction of hypernatraemic dehydration is very difficult. Among various schools of thought about the choice of fluids, ORS is preferred for a slow and gradual restoration of the deficit because oral or nasogastric (NG) rehydration using ORS is safe and associated with significant reduction in complications compared to intravenous rehydration (10,11). Correction of hypernatraemic dehydration using intravenous hypo- or hypertonic solution is often used in preventing a rapid fall of serum sodium and consequent cerebral oedema. However, there are no conclusive data on the efficacy of the use of either ORS or intravenous hypo- or hypertonic solution for the safe correction of hypernatraemic dehydration.

The Dhaka Hospital of icddr,b admits a large number of diarrhoeal patients, and a number of them also present with hypernatraemic dehydration. Most of the patients receive only ORS for the correction of hypernatraemic dehydration, irrespective of the level of serum sodium. A report of a patient whose serum sodium was 201 mmol/L and received only ORS for correction, is presented here.

## CASE HISTORY

A boy aged 4 months 7 days from a middle-class family (monthly family-income of Tk 10,000-15,000 equivalent to US dollar 121-181) living in Dhaka district was brought to the Dhaka Hospital of icddr,b on 9 January 2011 and admitted to the Short Stay Unit on the same day. Immediately after admission, he was shifted to the hospital's ICU for his respiratory distress and irritability. He had a history of watery diarrhoea for 3 days, associated with vomiting for the same duration, fever and respiratory distress for the last 12 hours. His stool frequency was 12 times/day, vomiting 2 times/day, and fever was high and continued but not associated with chills and rigours, and respiratory distress was not associated with cough. The child had passed urine half-an-hour before admission in the ICU. He received 6 packets (3 litre) of inappropriately-prepared (concentrated) ORS at home but no drugs. History of his past illness was unremarkable. He did not have a history of contact with any persons known or suspected to have tuberculosis, and his family members were in good health. He was the second and the last issue of his non-consanguineous parents and was delivered normally at home at full term. His weight and height at birth could not be obtained. He was breastfed at birth, and it continued up to 2 months. Since then, he was given mixed-milk (formula milk and breastmilk). His developmental milestones were age-appropriate, and his vaccinations were up-to-date according to the local EPI (Expanded Programme on Immunization) schedule. Both the parents were literate (education of the father and the mother was up to college and high school level respectively); the father was a garments worker, and the mother was a housewife. They were living in a tinshed house, used to drink tubewell water but their sanitation was poor.

On admission, the infant was initially irritable and excessively thirsty but he became very lethargic soon after admission. His pulse rate was 146 per minute with normal volume, blood pressure was 85/60 mmHg, capillary refill time was <2 sec, rectal temperature was 39.5 °C, and respiration rate was 80 per minute. He weighed 6.4 kg and had a length of 60 cm; his anthropometric measurements showed a z-score of <-2 [>-3 of the median of WHO growth charts] for all of the three indices—weight-for-age, weight-for-length, and length-for-age. He had some dehydration but did not have pallor, cyanosis, jaundice, oedema, or clubbing.

There was no increased work of breathing (no marked use of accessory muscles of respiration, and he did not have nasal flaring, head-nodding, stridor, wheezing, grunting respiration, or lower chest-wall in-drawing). His trachea was centrally placed, and the respiration sounds were vesicular with no adventitious sounds (rales, rhonchi, or pleural rub). Other systemic examinations of the infant were not remarkable. His blood glucose, measured at bedside, was 7.30 mmol/L, and his arterial oxygen saturation (SPO_2_) was 96% in room-air (WHO defines SPO_2_ of <90% at sea-level as hypoxaemia).

Thus, the initial problems were: (a) acute watery diarrhoea with some dehydration, (b) hypernatraemia (on the basis of history and cardinal clinical signs), (c) pneumonia (according to WHO definition), and (d) lethargy (differentially thought to be due to hypernatraemia itself, and also sepsis).

The laboratory work included: blood for total and differential white blood cell (WBC) count; blood culture and sensitivity; serum electrolytes, including serum total calcium, total magnesium, serum creatinine; rectal swab culture for isolation and identification of enteric pathogens; urine for routine and microscopic examinations (RME); and urine for culture and sensitivity (as a part of routine investigations in a febrile infant); and a chest x-ray.

We started management of dehydration with ORS following WHO guideline (12). Parenteral ceftriaxone and levofloxacin was also started to cover aetiologic agent in pneumonia and probable sepsis. Feeding was initiated according to standard protocolized management of diarrhoea.

His total WBC was 9,600/mm^3^, with 55.1% neutrophils, 0.0% bands, 33.5% lymphocytes, 11.2% monocytes, 0.1% eosinophil, and 0.1% basophil. Serum sodium was 201.0 mmol/L, chloride 165.3 mmol/L, potassium 3.6 mmol/L, TCO_2_ 20.3 mmol/L, total calcium 1.94 mmol/L, total magnesium 1.31 mmol/L, and serum creatinine 66.6 micromol/L. The chest x-ray and urine RME revealed normal findings.

After receiving the electrolyte report (around 4 hours after initiation of hydration), we re-evaluated the dehydration status and found no signs of dehydration. Then we decided to manage the correction of hypernatraemic dehydration, using ORS only. The volume required was calculated following a formula [10÷(molecular concentration of sodium in a given solution–measured serum sodium)/(0.6×weight in kg+1)=litre/24 hours] (2) aiming not to reduce the serum sodium by >10 mmol/L/24 hours. We also maintained hydration by replacing the ongoing loss of each purging. ORS was administered through a nasogastric tube. In spite of having his dehydration under control, the patient was still experiencing fast breathing, indicating pneumonia even in absence of radiological evidence (13).

Correction of hypernatraemia was started with a calculated rate of ORS of 15 mL per hour through nasogastric tube, and the correction of hypocalcaemia was tried with 10% calcium gluconate injection (0.5 mL/kg), and oral supplementation with calcium carbonate tablet was continued during hospitalization. Ten hours after initiating the treatment of hypernatraemia (14 hours after admission), the patient developed a generalized tonic clonic seizure. The seizure was difficult to control and required a total of three doses (0.1 mg/kg/dose) of injection lorazepam (10 minutes apart), followed by a loading dose of injection phenobarbitone (20 mg/kg) and injection phenytoin (20 mg/kg). Simultaneously, a maintainance dose of phenobarbitone (5 mg/kg/day in two divided doses) and phenytoin (2.5 mg/kg/dose 12 hourly) were continued to prevent further convulsions. After cessation of the convulsion, we re-examined the child and did not find any signs of meningism except extreme lethargy. We differentially thought that convulsion was either due to hypernatraemia itself or due to cerebral oedema from a probable rapid fall in sodium. The serum sodium level after 24 hours was 185 mmol/L, and the fall was >0.5 mmol/L/hr. We examined the fundi and did not find any evidence of cerebral haemorrhage or cerebral oedema. However, there was another episode of generalized tonic clonic convulsion which was not controlled by an additional dose of injection lorazepam (0.1 mg/kg/dose), and an empirical dose of 20% mannitol injection [0.5 g (2.5 mL)/kg/dose] was given due to the likelihood of cerebral oedema; the convulsion was controlled after infusion of mannitol. We gave another dose [0.5 g (2.5 mL)/kg/dose] of mannitol 6 hours after the initial dose. We re-examined the fundi and still did not find any evidence of cerebral oedema and, therefore, stopped mannitol. Then approximately every 24 hours, the serum sodium was checked (which was 174 mmol/L, 157 mmol/L, 152 mmol/L, and 145 mmol/L), and the volume of ORS needed for the correction of hypernatraemic dehydration was re-calculated each day. On average, the fall was <0.5 mmol/L/hr for the next 4 days during the correction. In the mean time, by day 3, diarrhoea was controlled, and clinical signs of pneumonia disappeared but the patient was still lethargic. The lethargy might have been due to the use of two anticonvulsants and as there were no further episodes of convulsion, the anticonvulsants were weaned after a convulsion-free period of 48 hours; all the anticonvulsants were gradually weaned successfully by day 7. On day 7, the patient was active and alert, took food orally and was gradually becoming afebrile. There were no CNS abnormalities. In the mean time, no organism was isolated from blood, rectal swab or urine culture, and we stopped antibiotics and planned to discharge the patient.

**Fig. 1.**
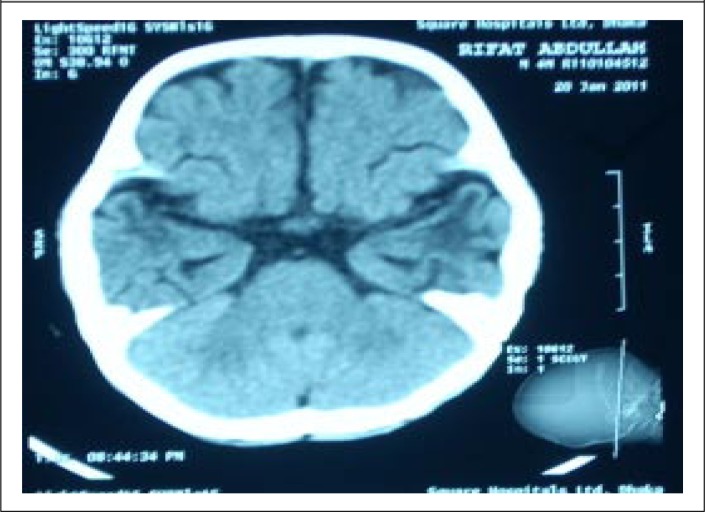
CT scan: Bilateral low-attenuation areas are seen in both temporal poles, overlying cortex is spared

**Fig. 2.**
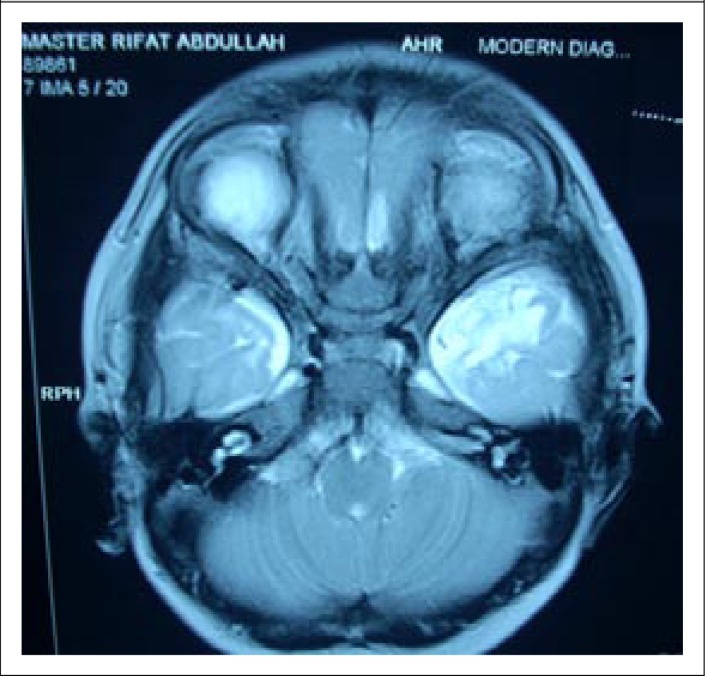
MRI scan: T2 hyper-intense area is seen in the left temporal pole

Before discharge (on day 12 of his hospitalization), a computed tomography scan of brain was performed, which revealed mild ischaemic changes in both the temporal lobes (Fig. 1). After review by a paediatric neurologist from a local tertiary-care centre, a Magnetic Resonance Image (MRI) scan was performed, which revealed ischaemic changes in left temporal lobe (Fig. 2), and this was consistent with the CT scan findings. The patient has been under regular follow-up at our hospital and at the last follow-up on 15 November 2011, the patient was found to be playful, and his developmental curve was up-to-date. However, there was a history of occasional episodes of absence seizure persisting for a few seconds over the last two months.

## DISCUSSION

We have a few distinct observations from this case study. This febrile young infant with acute watery diarrhoea had the highest serum sodium reported in the literature that was successfully managed using only ORS, though we had difficulty in controlling seizure during the initial correction.

The presence of 201 mmol/L of serum sodium was probably due to a combination of several factors, including early infancy, taking concentrated ORS after each purging, high fever, pneumonia with fast breathing, and probably having rotavirus diarrhoea—the most common pathogen during the winter season. Taking concentrated ORS gives rise to hyperosmolality of the extracellular fluid, causing hypernatraemia, simultaneously leading to the movement of water from the intracellular fluid (ICF) to the ECF, resulting in intracellular dehydration and excessive thirst (14,15). In Bangladesh, early January is within the winter season while the main aetiologic agent in diarrhoeal stool is rotavirus (16,17). Although rotavirus was not isolated from this case as it was not part of the routine workup in our ICU, hospital surveillance data among children below two years of age during the same period (January 2011) revealed the highest incidence (67%) of rotavirus diarrhoea (personal communication, Faruque ASG, 2012). Moreover, no other aetiologic agents could be isolated from the stool. The faecal loss of sodium in rotavirus diarrhoea is often below 40 mmol/L (18,19) whereas the molecular concentration of sodium in the reduced-osmolarity ORS available in the market is 75 mmol/L (12,20). This is quite high for the hydration of a patient with rotavirus diarrhoea, and the use of ORS in an appropriate concentration and volume might give rise to hypernatraemia. Moreover, in winter, the low humidity in the atmosphere causes increased insensible loss of water in patients, especially in young infants, contributing to hypernatraemic dehydration (5,21). These factors might have an impact on the extreme hyprnatraemia in this case. High fever and fast breathing with pneumonia were responsible for the evaporative loss of water that occured through lungs, skin, and urine in this infant (5,21). Young infants have a larger surface area in relation to height or weight than adults; they lose relatively more water by evaporation, especially when cared for on radiant warmers which may raise the level of hypernatraemic dehydration (5,21).

Our observation of the successful use of ORS through a nasogastric tube for the slow correction of hypernatraemic dehydration in this infant might be the secret for the successful correction of extreme hypernatraemia. The formula that we have used for the correction of hypernatraemic dehydration has been well-tested in adults (2) but this is the first time that the formula was tested in an infant, using only ORS with such level of serum sodium. The infant experienced an initial setback with the development of seizure that was hard to control. This might be due to either hypernatraemia itself or cerebral oedema developed during the correction of hypernatraemic dehydration. Hypernatraemia causes hyperosmolar ECF and shift of ICF to ECF, leading to intracellular dehydration and potentially causes cerebral haemorrhage and convulsion (4,5,15). On the other hand, rapid correction of hypernatraemic dehydration in this case might cause a rapid fall in ECF osmolality due to rapid shift of serum sodium into cells. A fall in the concentration of other osmotically-active substances such as glucose may lead to excessive movement of water into cerebral cells, leading to cerebral oedema as well as haemorrhage potentially responsible for the development of seizure (3-5,10,22). However, we did not have any clinical evidence of cerebral haemorrhage or cerebral oedema, although both CT scan and MRI were consistent with left temporal lobe ischaemia. Although the convulsions in our patient were finally controlled by the management of cerebral oedema, we are not quite sure whether the left temporal lobe ischaemia was due either to the hypernatraemia itself or due to cerebral oedema caused by the rapid fall of serum sodium. However, we stopped mannitol after just 2 doses, and it is very unlikely that cerebral oedema with such uncontrolled seizure would be controlled only with the two doses of mannitol. The logical use of multiple anticonvulsants might have had a real impact in ultimately controlling the seizure. On the other hand, a rapid fall in serum sodium is usually associated with a high case fatality but in this case, the patient survived, became playful, and remained well after discharge, although he developed episodes of absence seizure persisting only for a few seconds 9 months after discharge. This suggests that the hypernatraemic dehydration might have developed acutely and potentially warranted rapid correction (fall of serum sodium was at a rate of 1 mmol/L/hr) (8,9). However, the scrupulous adherence to the use of ORS following the above formula is safe for the successful correction of hypernatraemic dehydration. The safe use of ORS for the correction of hypernatraemic dehydration has also been reported previously (7,23).

The child might develop problems of speech perception, with difficulty in discriminating speech in advancing age but young children have an ability to let the remaining part of the brain take over the function of a damaged part (24). The child will be kept under regular follow-up by a paediatric neurologist to keep a close eye on his speech development.

### Conclusions

The use of only ORS for the correction of extreme hypernatraemia in a young infant has been found to be very successful. Thus, scrupulous adherence to the use of ORS in such a case may be the choice for the management of hypernatraemic dehydration. However, before making a final recommendation, a randomized clinical trial to evaluate the efficacy of ORS compared to intravenous solution is imperative.
